# Breast Cancer Detection on Histopathological Images Using a Composite Dilated Backbone Network

**DOI:** 10.1155/2022/8517706

**Published:** 2022-07-06

**Authors:** Vinodkumar Mohanakurup, Syam Machinathu Parambil Gangadharan, Pallavi Goel, Devvret Verma, Sameer Alshehri, Ramgopal Kashyap, Baitullah Malakhil

**Affiliations:** ^1^Bell, Montreal, Canada; ^2^General Mills, 220 Carlson Parkway, Apt 208, Minnetonka 55305, Minnesota, USA; ^3^CSE, FET, MRIIRS, Faridabad, India; ^4^Department of Biotechnology, Graphic Era Deemed to be University, Dehradun, Uttarakhand, India; ^5^Department of Pharmaceutics and Industrial Pharmacy, College of Pharmacy, Taif University, P.O. Box 11099, Taif 21944, Saudi Arabia; ^6^Amity University, Raipur, Chhattisgarh, India; ^7^BCS Faculty, Rana University, Baraki Square, Kabul, Afghanistan

## Abstract

Breast cancer is a lethal illness that has a high mortality rate. In treatment, the accuracy of diagnosis is crucial. Machine learning and deep learning may be beneficial to doctors. The proposed backbone network is critical for the present performance of CNN-based detectors. Integrating dilated convolution, ResNet, and Alexnet increases detection performance. The composite dilated backbone network (CDBN) is an innovative method for integrating many identical backbones into a single robust backbone. Hence, CDBN uses the lead backbone feature maps to identify objects. It feeds high-level output features from previous backbones into the next backbone in a stepwise way. We show that most contemporary detectors can easily include CDBN to improve performance achieved mAP improvements ranging from 1.5 to 3.0 percent on the breast cancer histopathological image classification (BreakHis) dataset. Experiments have also shown that instance segmentation may be improved. In the BreakHis dataset, CDBN enhances the baseline detector cascade mask R-CNN (mAP = 53.3). The proposed CDBN detector does not need pretraining. It creates high-level traits by combining low-level elements. This network is made up of several identical backbones that are linked together. The composite dilated backbone considers the linked backbones CDBN.

## 1. Introduction

Breast cancer treatment is a multidisciplinary endeavor, with the pathologist's role being to determine the correct diagnosis. To detect a cancerous breast growth, anomaly screening imaging, pathological imaging, mammography, magnetic resonance imaging, and ultrasound were used. The type of radiographic imaging or histopathological images used is determined by the characteristics of the breast tissue. A radiographic deviation from the norm reveals a tissue test that must be obtained to make the correct judgment. The pathologist evaluates the tissue to determine whether it is beneficial or harmful (malignant). Not all masses seen by imaging represent malignant development. Certain bosom lumps, such as fibroadenomas, are benign tumors [1]. A biopsy involves the use of a small needle to extract the thin center of the tissue, which is subsequently processed in a pathology lab. The tissue is divided into small parts and placed on glass slides. Under the magnifying lens, the striking colors imparted to the glass slides that stain the tissues pink and blue are visible. Pathologists examine the slides with a magnifying glass to evaluate if the tissue test is for a hazardous malignant growth. Creating many similar backbones may result in a stronger backbone for cancer detection. The composite dilated backbone (CDB) considers the linked backbones. This network is made up of several identical backbones that are linked together, known as helper and lead backbones. Composite connections transfer higher-level characteristics to the next backbone layer from left to right, while the lead backbone's feature maps are utilized for object detection. CDBN improves detection performance by combining high-level and low-level data from many backbones [2]. The visible layer of CDBN receives random overlapping white images. Minibatches are taught through pictures. When a sample is not representative, they assist in reducing the noise. It creates high-level traits by combining low-level elements. Notably, unlike a pretrained model utilizing ResNet or ResNeXt, a CDBN detector does not need pretraining. Before one does that, create a new backbone and train it on ImageNet.

A backbone network trained on the ImageNet dataset is often used to extract core object recognition attributes. It seems to be rational since more representational features suggest higher performance. Despite encouraging results from improved detectors with deep and broad backbones, further study is needed [3, 4]. Pretraining a new backbone on ImageNet improves detection performance as well. When gathering basic item identification data using contemporary backbone networks, a similar problem develops. In the BreakHis dataset, we obtain 53.3 mAP for object identification.

In Section 2, we describe how to generate many extremely comparable object-detecting backbones, which may improve the performance of many existing detectors.  Section 3 represents the proposed CDBN model, Section 4 displays experimental results, and Section 5 gives the conclusion.

## 2. Background

Many convolutions neural networks (CNNs) have helped with the categorization and detection of the items described here to train deep CNNs faster.

### 2.1. AlexNet

**Figure 1:**
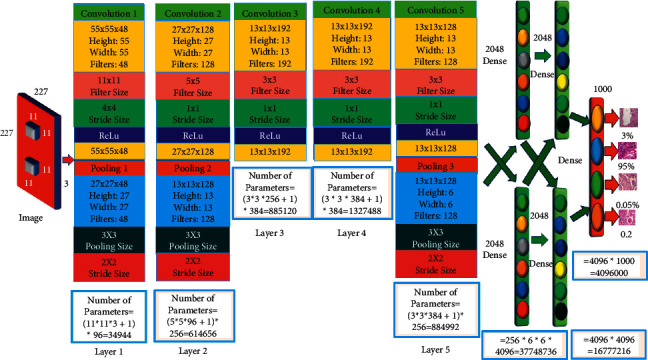
Internal processing of AlexNet architecture.

AlexNet recommends utilizing saturating activation functions, such as Tanh Sigmoid [5]. Overfitting is reduced by dropping out, while feature augmentation is corrected by max-pooling. AlexNet improves training efficiency. The number of parameters in the AlexNet's movement dataset has been raised to 62.3 million. The picture measures 227 × 227 × 3. It uses 4096 neurons to classify 1000 objects. The image was convoluted using 96 11 × 11 kernels, stride size 4, max pooling with stride size two, and filter size 3 × 3. The convolution with 128 filters of size 5 × 5 and a stride is used to create 4096 neurons. Then, the convolution with 192 filters and one stride followed by max-pooling with 128 filters of stride two and filter size 3 × 3 were used. The Softmax activation function gives 1000 values together with classification scores. As indicated in the internal architecture of AlexNet in Figure 1, the number of trainable parameters is 34,944, 614,656, 885,120, 1,327,488, 884,992, 37,748,736, 16,777,216, and 4,096,000, for a total of 62,369,152 parameters.

### 2.2. VGG-16 Network

**Figure 2:**
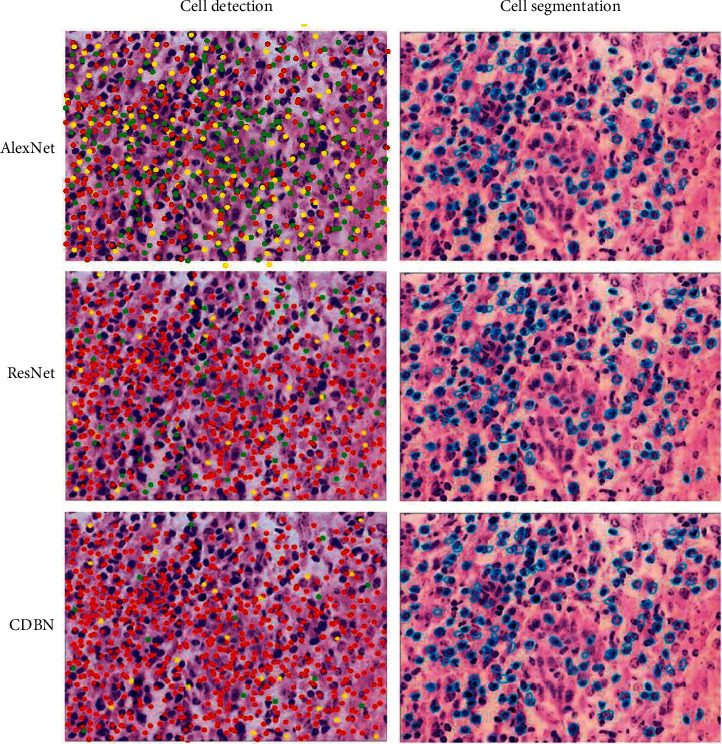
Different techniques of diseased tissue cell identification and segmentation in database B (TP in red, and green was FN, with a blue dividing line).

It accepts 224 × 224 images as input and has three channels, which are ensured by two 64-kernel convolutions in layer 1. The max pool layer with a stride of 2 × 2 reduces the dimension by 50% with 128 channels, followed by two convolution layers in layer two. Layer 3 is made up of a pooling layer, three 256-channel convolution layers, layer four and layer five, max-pooling, and three 512-channel convolution operations. Layer 6 consists of two totally connected layers of 1 × 1 × 4096 neurons, followed by a set of 1000 neurons. These are sent to the SoftMax layer, which generates the classification values. ReLu is used in VGG-16 hidden layers, such as AlexNet, to make VGG-16 more efficient and faster to train.  Figure 2 depicts the number of trainable parameters required in each layer to be 36,844, 147,456, 589,824, and 2,359,296. It takes 102,760,448, 16,777,216, and 4,096,000 trained parameters for the completely connected layers, for a total of 138 million parameters. The VGG-16 network has 16 layers and an error rate of 7.7 percent. This network uses a real-time activation algorithm without local response normalization to conserve memory [6, 7]. The VGG employs a 3 × 3 kernel with stride 1, while AlexNet employs an 11 × 11 kernel with stride 4. Max-pooling decreases the picture size after five convolution units. In the first layer, there are 4096 neurons. The second layer reduces the picture size while increasing the number of channels in the picture.

### 2.3. ResNet (Residential Network)

Gradients are dropped in the deep layers as the network depth and learning rate are changed. ResNet, which is based on residual networks, addresses the learning rate issue by introducing a network layer that boosts performance [8]. ResNet addresses the problem by bypassing the input information to the output while retaining the integrity of the information. The network is trained on input and output changes. ResNet varies from other networks because of bypassed or skipped connections [9]. Some layers in the deeper network may be ignored by the ResNet, causing the network's output to vary. It is as follows: in the layer, the input and weight are multiplied, and bias is added.

**Figure 3:**
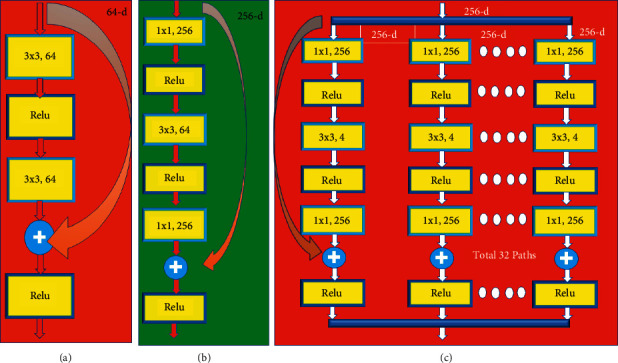
(a) A basic ResNet block. (b) A bottleneck block for ResNet-50/101/152. (c) RestNext 50 building block with cardinality 32.

**Figure 4:**
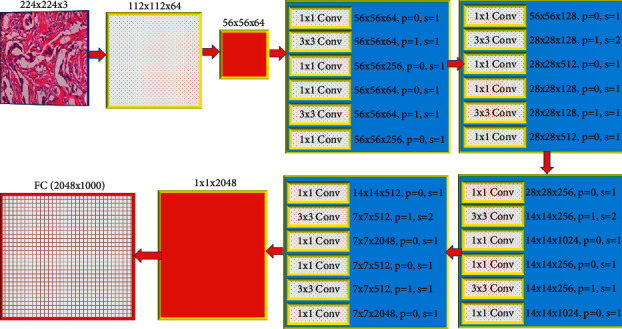
The architecture of 50 layers ResNet.

Change the number of deep layers and their position, which is amazing. 11.17 million variables and 512–64 output channels are available. ResNet 152 contains sixteen routes shown in Figure 3, while ResNet 50 has 32 shown in Figure 4. With 128–2048 ResNet-152 filters, it made more than 20% of the top-1 and 5 errors. The abbreviation for 64 broad routes is 64 × 4 d. The main distinction is between flaws in training and errors in testing.

Table 1 shows a comparison of benign, intermediate, and malignant phyllodes tumors with attributes like tumor edge, connective tissue cells, mitoses, atypia of stromal cells, overgrowth of the stroma, and heterologous cancerous elements. It has less than 25% glandular tissue and stroma. The glandular tissue and stroma account for 25–50% of the glandular tissue and stroma in the Level 2 scattered fibro glandular bosom. The glandular tissue stroma contributes to about 75% of the very thick breast tissue because of the abundance of adipose tissue. Bosom thickness levels 1 and 2 are referred to as “nonthick.” As the fat shows black on a mammogram while the disease appears white, having more fat in level 1 and level 2 thickness bosom facilitates the identification of bosom cancer.

A white tumor on a dark backdrop is difficult to spot because the glandular tissue, the stroma, and tumors are all white on a mammogram. The abundant glandular tissue and stroma in level 3 and level 4 bosoms make detecting malignant development more challenging. Therefore, it is more difficult to recognize a white disease against a white backdrop. High breast thickness makes the bosom feel harder and uneven, making physical inspection more difficult to discover a tumor. Improved mammography is currently the most effective technique to screen for breast illness, however, women with dense breasts may wish to consider alternative imaging procedures to detect the signals of malignant breast growth early on [10–14].

Mitoses that are cellular, moderate, and asymmetrical, when analyzing phyllodes tumors, precision and consistency are necessary. It is vital to correctly diagnose potentially deadly phyllodes tumors, which must then be carefully removed and appropriately treated. The risk of metastasis and mortality from these tumors is well-recognized, yet it is quite varied. Subjectivity and other human variables, however, may lead to mistakes that affect tolerant consideration. Given the significance of these advancements in the promising field of computerized pathology, the cycle is also time-consuming and costly. There are many distinct forms of breast tumors that may be classified based on how the tumor cells appear under a microscope. These categories apply to both benign and malignant tumors.

## 3. The Proposed Model

**Figure 5:**
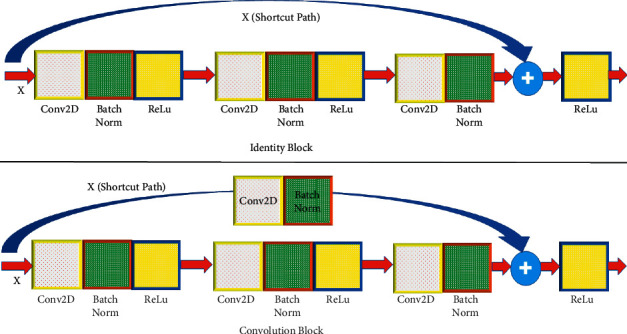
The identity and convolution block.

The histopathology breast image is provided below in the RGB (color) format. The RGB format makes it more difficult to distinguish between cell nuclei and other components. Preprocessing smoothens the pixels and boosts the contrast. Smoothen the picture using the median filter. Bottom-hat and top-hat filtering reduce noise in images. Increase the contrast between the nucleus of the cell and the background. The median filter, which is a nonlinear filter, successfully retains cell edges in the images of breast cancer. The purpose of feature extraction is to enhance the overall classification and prediction performance [15, 16]. The process involves producing prospective features using different transformation techniques. These attributes are put together to make feature vectors, which are then used as inputs in the classification process. ResNet is split into two sections. The first block is the identity block, which has the same input and output dimensions as the other blocks. The second kind is the convolutional block, which is useful when the size fluctuates, as seen in Figure 5, which is used in the proposed model along with AlexNet to get accurate results. When the input and output activation have the same dimensions, the identity block is a frequent ResNet block to utilize.

The original measurement variable function is used to categorize and recognize patterns. This subset is chosen by a genetic algorithm. The classifier analyses this data to determine the picture's benignity. These are overfitting metrics. The genetic algorithm employs random people's genes to optimize. BreaKHis is categorized into two categories: helpful tumors and dangerous tumors. Unlike previous needle biopsy techniques, this one is conducted in an emergency clinic under general anesthetic. Fibroadenomas are most frequent in women in their twenties and thirties. Malignant phyllode tumors are a rare kind of breast cancer. Lobular carcinoma occurs in the groupings of milk-producing organs called the lobules. LCIS is neither a real precancerous condition nor a benign cervix growth. Papillary carcinoma (P.C.) malignant growth is the most frequent kind of thyroid disease. Mucinous carcinoma may occur in places other than the bosom, such as the colon or rectum. Deep learning is employed by the model to recognize and categorize photographs accurately. Maximum pooling is a downsampling process used to reduce the size of a picture, thus saving calculation time and preventing overfitting. ReLu avoids the cancellation problem because of the zero-threshold value. It does not need any learning parameters, and the channel size remains constant throughout the processing. The bulk of target detectors in the market today are based on image categorization. This work proposes a novel method for combining existing backbones to create a new backbone that is just for target identification. CDBN combines advanced and low-level capabilities from many backbones, resulting in increased detection performance. A hidden layer is added to the input layer of the model.

Reduced overfitting, increased model speculation, and improved performance are the advantages of information expansion. As histological imaging datasets for breast cancer are scarce, overfitting occurs in CNNs when the models absorb the complexities of data preparation too effectively. The recommended method of adding new data is time-and money-consuming, however, it overcomes the issue of overfitting.

They pay special attention to two major aspects: overall design and cytological details. The photographs are classified by these professionals based on the kind of bosom ailment and the amount of damage within each group. Deep learning is employed by the model to recognize and categorize photographs accurately [17–20]. By looking at the hematoxylin and eosin-bloated tissue, pathologists can figure out the kind of malignancy and how bad the cancer is.

CLAHE lowers noise amplification and enhances neighborhood discrimination by restricting the height of the neighborhood histogram. This method separates the picture into subareas before rearranging the histogram of each subdistrict. Then, independently level each subdistrict's histogram, gain the changed dark incentive by adding each pixel, and see the difference limited to flexible histogram even with image improvement [21, 22]. Make the image in a more detailed manner, and make the edges sharper in every part of it. CLAHE analyzes individual tiles rather than the whole picture. Bilinear interpolation is used to eliminate ambiguous tile borders. Using this method will boost the contrast of images.

As it performs better than tanh and sigmoid, ReLu (rectified linear unit) substitutes the negative value after convolution with zero. The ReLU layer of a convolutional neural network combines nonlinearity with rectification. One has no control over the right side, and the threshold is set to zero. It is a piecewise model that performs basic computations.

(1) $$\begin{array}{c} C_{i}^{L}=\max\left(0,\;C_{i}^{\left(L-1\right)}\right). \end{array}$$

Equation (1) shows the ReLU function. The ReLU function, which is the function's derivative, is likewise monotonic in nature. In contrast, the output of *C*_*i*_^(*L* − 1)^ is 0 for any negative input, whereas the output of *C*_*i*_^(*L* − 1)^ is any positive input. Because of this, the output is a value that is somewhere between 0 and infinity. After examining how the ReLU activation function modifies inputs, the next step is to visualize the resulting changes in the data.

Firstly, start by creating the ReLU function, so that one knows what one is talking about.

  ReLU (*C*_*i*_^(*L* − 1)^)
  If (*C*_*i*_^(*L* − 1)^) > 0, then: *C*_*i*_^(*L* − 1)^
  else:
  return an empty (zero).

The positive value after the operation indicates that it is more significant, while the negative value indicates that it is less essential. The value greater than zero remains unaltered, however, the value less than zero is reset to zero. These are some of the reasons behind ReLu's exceptional performance. It reproduces the gradient productivity and avoids the cancellation problem because of the zero-threshold value. It is straightforward and efficient to implement in CNN.

Depending on the training data, each layer has between 50 and 700 neurons. CDBN is made of several identical backbone networks (divided into auxiliary and main backbone networks) and composite linkages that connect them. Using CDBN saves time over creating a new backbone network and pretraining on ImageNet. CDBN's key feature effectiveness has been enhanced. Foreground items exhibit greater activation levels than background objects according to the feature maps in our CDBN. They all rise by 1.5 to 3.0%, which shows that our composite backbone network works well. CBNet connects the parallel stages of neighboring backbones through composite connections, resulting in numerous similar backbones. It is used in object identification to feed the output features from the previous backbone into a new backbone, which then outputs the features from the backbone that came before that one.

The bulk of target detectors on the market today are based on image categorization. Detection is finetuned after pretraining using ImageNet. Then, there is the question of whether this kind of backbone can be utilized to extract image features for target identification to maximize performance [10, 23–26]. The expense of reaching exceptional detection performance, on the other hand, would be rather significant if a new backbone was developed and pretrained on ImageNet. Therefore, this work proposes a novel method for combining existing backbones to create a new backbone that is just for target identification.

**Figure 6:**
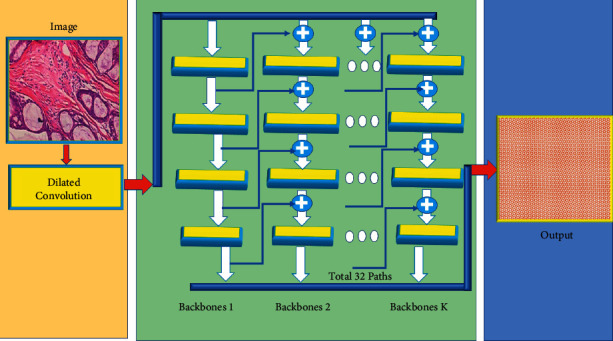
Composite dilated backbone network for object detection.

This study offers a CDBN as seen in Figure 6. The image after performing dilated convolution is given to the backbone network, and then the backbone network generates the features that are given to ResNet, followed by AlexNet that accurately detects the cancerous cells. It connects multiple stages with neighboring backbones at the same horizontal point, thereby connecting many stages [26]. The backbones have been merged. The diagram depicts the whole information flow from left to right. The output of the helper backbone (also known as advanced features) is sent through the composite connection to the next backbone, which acts as the stage's input at the same horizontal position, and the output of the final backbone (lead backbone) is used for target identification.

CDBN combines advanced and low-level capabilities from many backbones, resulting in increased detection performance. CDBN does not need to be pretrained. Instead, CDBN must employ pretrained backbone models (such as ResNet and ResNetXt) to create each backbone. Dilated convolution enhances receptive field while retaining feature map resolution (max-pooling or strided convolution will reduce the feature map resolution). The “dilation rate” hyperparameter is proportional to the convolution kernel's intervals. It reduces the conflict between feature map resolution and receptive field size, however, it has drawbacks. All neurons in a feature map have the same receptive field, allowing for a single scale. In other words, putting CDBN into use is more efficient and cheaper than making a new model.

## 4. Experimental Results

The efficacy of features enhanced through a composite dilated backbone network as compared to a single backbone network is shown in this section.

### 4.1. Datasets

The study used datasets BreakHis [23] and BreCaHAD [10]. In all, 82 individuals contributed 7909 photos of breast tumors (2480 benign and 5429 malignant) (40x, 100x, 200x, and 400x). BreCaHAD is a red, green, and blue microscopic picture collection.

### 4.2. Pathological Image Classification Using CNN

**Figure 7:**
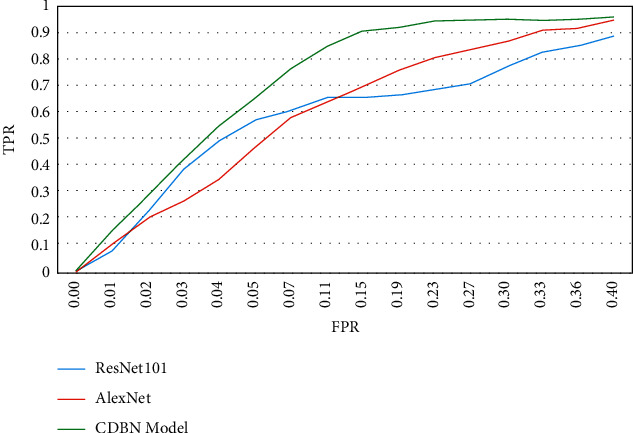
ROC curves for diseased tissue identification techniques.

The revised CNN algorithm's cell identification results were compared to those of ResNet101 and AlexNet in datasets. Across three datasets, the modified CNN approach had the greatest ROC AUC.  Figure 7 shows the graph between ResNet 101, AlexNet, and the CDBN model, and the parameters are true positive rate versus false-positive rate. It shows better and higher than 92 percent accuracy.

### 4.3. Pathological Cell Detection and Regional Segmentation Using CNN

**Figure 8:**
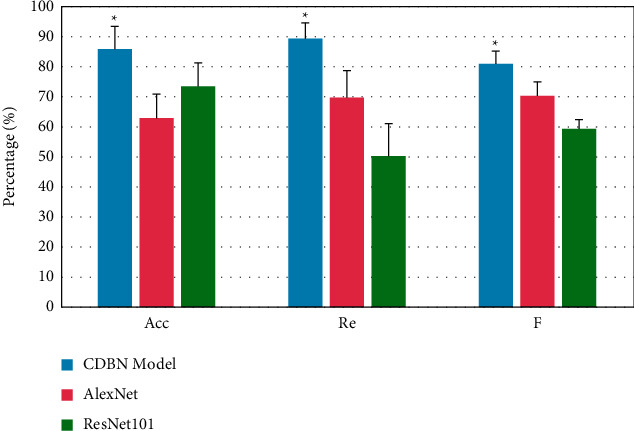
Comparison of abnormal cell identification performances by various algorithms.

In this study, the results of the CNN algorithm's aberrant cell detection were compared to the results of ResNet101 and AlexNet in Figure 8. Normal cell identification using the proposed model, ResNet101, and AlexNet model produced the averages of (86.05), (73.92), and (68.85), respectively.

The proposed model, ResNet101 model, and AlexNet model found ill cells with average *F* values of 81.99, 69.54, and 73.23. As indicated in Figure 8, these approaches discovered many problematic cells with average Re values of (88.43), (69.95), and (51.25). These results were higher than the other two methodologies' problematic cell identification rates of acc, *f*, and re (0.05). The ROR-PT image analysis accuracy averaged 76%. Kappa 0.47 ROR-PT was found to be more accurate in low-to-intermediate-grade tumors (85%) than in high-grade tumors (86%). Image analysis and histological subtype may predict tumor grade. Image analysis properly identified lobular and ductal cancers 94% of the time.

It was largely of a qualitative character. Many databases include the approach. Breast histopathology images were 85 percent accurate. 89.27 percent recalled and had an *F*-measure of 80.09 percent. The cells were then color-coded, with red representing TP and blue representing normal cells. Green is the color of the letter FN (missing cells). Misidentified cells were highlighted in yellow (FP), as shown in Figure 2. There was no global optimization applied in this study to recognize and segment images. The model's parameters will be modified later. The device counts the number of filamentous fractures. Breast cancer pathology photographs may assist in quantitative tumor grading. By detecting and segmenting cells in the pathology pictures of the breast tissue, the CNN algorithm aids in the detection and prognosis of breast cancer and other disorders. The feature graph accuracy of the two approaches grows with picture size. Stress histograms are outperformed by deep learning.

In this study, deep learning beats the standard stress histogram technique for extracting sick tissue features. There is a lot of promise in computer-aided diagnosis and prognosis in medical pathology. Our testing demonstrated a great influence now. There is still much to learn about pathological image analysis. In-depth research may still yield outstanding results in this field. A CDBN structure based on the conventional CNN structure completely accounted for multichannel image information and input picture scale. There are several advantages to using this methodology over the commonly used feature representation method. The workout tempo was also increased. Deep learning surpasses the traditional technique in this investigation. Parameterization and sampling with a base stress feature representation are still critical [34]. Parameters may be learned and compared.

## 5. Conclusion

Using dilated convolution, AlexNet, and ResNet, we developed a deep-deep learning-based model for breast cancer diagnosis. The composite dilated backbone network is an innovative method for integrating many identical backbones into a single robust backbone. We show that most contemporary detectors can easily include CDBN to improve performance. Creating a large number of similar backbones may result in a stronger backbone. CDBN improves detection performance by combining high-level and low-level data. Unlike a pretrained model utilizing ResNet or ResNeXt, a CDBN detector does not need pretraining. A backbone network trained on the ImageNet dataset is often used to extract core object recognition attributes. Pretraining a new backbone on ImageNet improves detection performance as well. On the BreakHis dataset, we obtain 53.3 mAP for object identification. CDBN is made of several identical backbone networks (divided into auxiliary and main backbone networks) and composite linkages that connect them. It is less expensive and more efficient to utilize CDBN instead of building a new backbone network and pretraining on ImageNet. Hence, it saves money and time. CDBN's key feature effectiveness has been enhanced. Foreground items exhibit greater activation levels than background objects according to the feature maps in our CDBN. They all rise by 1.5 to 3.0%, which shows that our composite backbone network works well. The histopathological images of breast tumors lack label information, leading to inaccurate grouping. To improve clustering accuracy, further study is required.

## Figures and Tables

**Table 1:** A comparison of benign, intermediate, and malignant phyllodes tumors.

Attribute	Benevolent	Marginal	Malignant
Tumour edge	Perfectly identified	Maybe centrally sneaky	Sneaky
Connective tissue cells	Similar to a noncancerous breast lump but often mild	Corpuscular and medium	Cellular and clear
Mitoses (high-power field)	<5 per 10	5–9 per 10	Greater than 10
Atypia of stromal cells	Nil or a little amount	Moderate to mild	It is possible to mark it
Overgrowth of the stroma	Missing	Missing or minor presence	Omnipresent
Heterologous cancerous elements	Missing	Missing	It is uncommon, however, if it is there, it is diagnostic.

## Data Availability

The study used datasets BreakHis and BreCaHAD which are available from the corresponding author. In all, 82 individuals contributed 7909 photos of breast tumors (2480 benign and 5429 malignant) (40x, 100x, 200x, and 400x). BreCaHAD is a red, green, and blue microscopic picture collection.
